# Physical activity fragmentation as a potential phenotype of accelerated aging

**DOI:** 10.18632/oncotarget.26631

**Published:** 2019-01-25

**Authors:** Amal A. Wanigatunga, Luigi Ferrucci, Jennifer A. Schrack

**Affiliations:** Department of Epidemiology, Johns Hopkins Bloomberg School of Public Health, Baltimore, Maryland, USA; Center on Aging and Health, Johns Hopkins University, Baltimore, Maryland, USA

**Keywords:** cancer, mobility, activity bouts, older adults, sensors

Though cancer is among the leading causes of death, death due to cancer is declining in the United States [1]. In 2016, the number of cancer survivors was estimated to reach 15.5 million and expected to grow to 20.3 million by the year 2026. Coupled with the rapid growth of the US older adult population, our society is faced with a looming challenge of maintaining health for an increasing number of cancer survivors.

Survivors of systemic cancers tend to exhibit aging phenotypes earlier in life—likely due to related surgical, radiation, and chemotherapeutic treatments [2]. Persons who have survived such treatments are likely to end up with systemic or regional organ tissue damage that increases the risk of cardiovascular and pulmonary disease along with second cancer onset. Further, symptoms of chronic idiopathic fatigue and pain are also common. Much of the underlying biological deterioration that has been connected to the damage from cancer and related therapies mimics the hallmarks of biological aging [2]. To date, much of the research on cancer and aging has been aimed at primary prevention, with exploration into long-term health effects just beginning to burgeon among cancer survivors. With advancements in measurement technology, detecting characteristics of accelerated biological aging among cancer survivors through novel phenotypes may better inform the development of effective strategies to optimize recovery from cancer sequela and treatments and possibly guide the selection and prescription of therapeutic regimens and behavioral interventions that maximize quality of life.

One of the strongest predictors of accelerated aging is an individual's ability to ambulate independently [3]. Mobility loss is associated with increased risk of many chronic diseases, such as cancer, cardiovascular disease, diabetes, obesity, arthritis, osteoporosis, as well as disability and premature mortality [4]. To gain contextual relevance of an individual's functional capability, self-report measures of ability to perform daily activities and participation in routine physical activity are commonly assessed. However, questionnaires are riddled with recall and social desirability biases, making it difficult to accurately measure the duration, frequency, and intensity of daily physical activities. Quantification of physical activity to this degree is important for pattern recognition of functional decline that is indicative of impending disability. The emergence of movement-based sensors, such as accelerometers, have made the measurement of at-home, free-living physical activity more feasible, and allow a deeper assessment of daily activity characteristics and patterns [5].

Accelerometers, which are fixed on a specific body location, typically collect accelerations at a sub-second level across multiple planes of space. With a high battery life and storage space, these monitors are used to continuously and non-invasively collect data for at least a week, yielding a large amount of data per participant. Yet, most large-scale studies that use accelerometry reduce these data into average means of total daily physical activity, or to estimate energy expenditure and time spent in different levels of physical activity. While such summary metrics are important, the rich and continuous movement data that are often available at the minute or second level are ignored.

Recently, the concept that physical activity during daily life can be subdivided into patterns of active and inactive (sedentary) bouts to provide important health information has gained momentum. Wanigatunga, et al showed that a physical activity intervention in older adults at risk for disability was effective in increasing time in short and long activity bouts across increasing physical activity intensities [6], but did little to reduce the impact of prolonged sedentary bouts [7]. Schrack, et al extended the concept of bouts by using minute-by-minute accelerometer data to derive the probability of transitioning from an active to a sedentary state throughout the day [8]. Collectively, this activity pattern measured through the probability metric characterizes an individual's “activity fragmentation”, which is a function of chronological age, fatigability, and poorer physical function, particularly at higher levels of functional capability.

**Figure 1:**
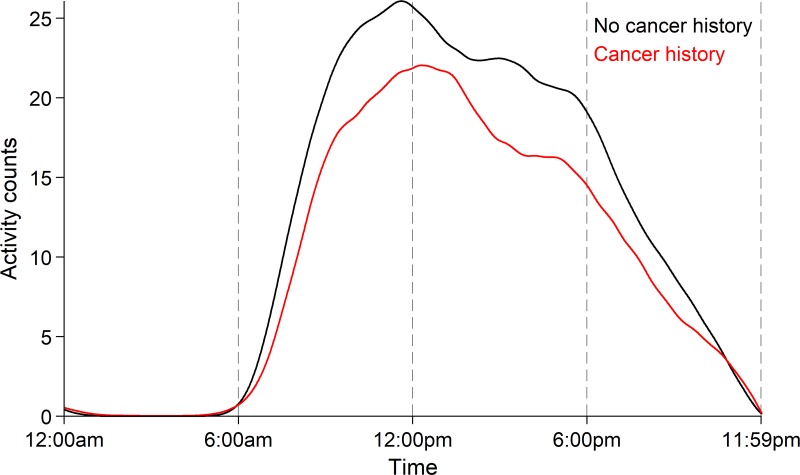
Median activity counts by cancer history status over 24 hours Deteriorating and diminishing diurnal patterns of physical activity cannot be captured via summary measures such as total time spent in physical activity. Fragmentation captures the cycling of physical activity and sedentary states that are performed throughout the day, providing a novel metric that quantifies the way daily physical activity is accumulated.

In Wanigatunga, et al's recent publication, activity fragmentation was coupled with total physical activity amount to create a physical activity phenotype that was assessed among older adults participating in the Baltimore Longitudinal Study of Aging by cancer history [9]. Together, the joint effects of physical activity quantity and accumulation patterns detected greater differences in physical activity than each independent characteristic alone, suggesting the combined physical activity phenotype may be more sensitive for detecting emerging physiological impairment resulting from cancer and related therapeutics. To this end, Figure 1 illustrates diurnal activity patterns in adults aged 50 and older, stratified by cancer history. The cancer history group has lower overall physical activity at all times throughout the day, with the cancer group showing a later activity peak than the group with no cancer history. Further, the cancer survivors' activity levels drop noticeably more during the afternoon until both groups equalize around bedtime. These differences are primarily driven by those whose physical activity is accumulated in a fragmented manner (e.g., take more breaks from being active), suggesting a diminished functional phenotype, at greater risk of high fatigability and poor endurance capacity [10].

These findings highlight important changes in both total daily activity and its accumulation patterns after cancer and related treatment, which may contribute to a higher risk of disability and reduced quality of life with aging. Possible underlying mechanisms remain to be defined but may include changes in energetic reserve through mitochondrial dysfunction and increased oxygen byproduct circulation accompanying chronic inflammation (“inflammaging”), and muscular deconditioning and atrophy driven primarily through sarcopenia. More research is needed to uncover the underlying biology contributing to activity fragmentation and to investigate complementary mechanisms such as the psychological (e.g., anxiety when faced with stair climbing at the grocery store) and the ecological (e.g., not walking to the grocery store because of the perceived obstacle of stair climbing) components to behavior-based activity movements. With the growth of data from accelerometers and wearables at both the research and commercial levels, innovative new measures of daily activity—such as activity fragmentation—are needed to advance aging-related research by providing novel, sensitive features of physical activity.
